# A Histopathological Study of Skin Lesions in Individuals with Oculocutaneous Albinism in Togo in 2019

**DOI:** 10.1155/2020/2361957

**Published:** 2020-07-29

**Authors:** Bayaki Saka, Julienne Noude Teclessou, Sefako Abla Akakpo, Piham Gnossike, Kwamé Doh, Saliou Adam, Abas Mouhari-Toure, Garba Mahamadou, Panawé Kassang, Yvette Elegbede Moise, Tchin Darre, Koussake Kombate, Palokinam Pitché

**Affiliations:** ^1^Service de Dermatologie et IST, CHU Sylvanus Olympio, Université de Lomé, Lomé, Togo; ^2^Service de Dermatologie et IST, CHU Campus Université de Lomé, Lomé, Togo; ^3^Centre National de Dermatologie de Gbossimé, Lome, Togo; ^4^Unité D'histopathologie, Clinique Mélia, Lomé, Togo; ^5^Service de Chirurgie Maxillo-Faciale et Plastique, CHU Sylvanus Olympio, Université de Lomé, Lomé, Togo; ^6^Service de Dermatologie et IST, CHU Kara, Université de Kara, Kara, Togo; ^7^Service de Dermatologie et IST, Centre Hospitalier Régional de Tomdè, Kara, Togo; ^8^Laboratoire D'anatomie et Cytotologie Pathologique, CHU Sylvanus Olympio, Université de Lomé, Lomé, Togo

## Abstract

**Objective:**

The aim of this study was to study the histopathological patterns of skin lesions in persons with albinism (PWA) in Togo in 2019.

**Method:**

During two mobile skin care clinics in 2019, biopsies/excisional biopsies were performed in PWA in case of clinical doubt or in front of lesions suspected to be cancerous for histological examination. Anatomopathological reports were thus analysed.

**Results:**

During the two mobile skin care clinics, 115 biopsies/excisional biopsies were carried out in 79 PWA, with a mean age of 24 ± 16.1 years. Histological examination led to a diagnosis in 110 cases (95.6%) and was inconclusive in 5 cases (4 cases of uncertain histological diagnosis and one case of nonspecific histological lesions). Fourteen different histological diagnoses were made, with a frequency ranging from 0.9% (one case) to 26.9% (31 cases). The four most frequent diagnoses in descending order were basal cell carcinomas (BCCs) (31 cases; 26.9%), invasive squamous cell carcinomas (SCCs) or Bowen's disease (23 cases; 20%), keratosis (20 cases; 17.3%), and cysts (seven cases; 6.1%). The 54 skin carcinomas were diagnosed in 33 (41.8%) of the 79 patients who underwent skin biopsies/excisional biopsies. The BCC/SCC ratio was 1.3. No cases of cutaneous melanoma had been diagnosed.

**Conclusion:**

Skin cancers represent the main histological diagnosis in PWA (46.9%) in Togo in 2019. The pattern of cutaneous malignancies in PWA shows the same trend as that seen in Caucasians with a predominance of basal cell carcinomas.

## 1. Introduction

Oculocutaneous albinism is an inherited disease characterized by the total or partial absence of melanin in the skin, hair, and eyes [1, 2]. Melanin, the pigment responsible for skin colour, is photoprotective against carcinogenic ultraviolet radiation [3]. Its deficiency in people with albinism (PWA) predisposes them to the harmful effects of this radiation, with the development of actinic damage and a major risk of skin cancer [4–6]. The diagnosis of skin diseases in PWA very often requires histological examination [2, 7–9], especially in the case of skin lesions suspected of being cancerous. Indeed, the fear of the practitioner is the early detection of these skin cancers in this population because of their very high frequency, varying from 23 to 26% depending on the series [9–12]. We conducted this study in order to study the histopathological patterns of skin lesions in persons with albinism (PWA) in Togo in 2019.

## 2. Method

Two rounds of free mobile skin care clinics took place throughout the Togolese territory in 2019 in order to treat malignant and premalignant lesions in PWA. These campaigns covered 10 cities in Togo, the first in June and July 2019 and the second in October and November 2019. Dermatological consultations were provided by a senior dermatologist. Biopsies/excisional biopsies were carried out in case of clinical doubt or in front of lesions suspected of being malignant and sent to the laboratory of pathology of the Sylvanus Olympio University Hospital or to the histopathology unit of the Mélia clinic in Lomé for histological examination. Two pathologists assisted in carrying out the histological examinations. Anatomopathological reports were collected and analysed. The data collected were sociodemographic (age and sex) and histological diagnosis.

## 3. Results

During these two campaigns, 115 biopsies/excisional biopsies were carried out for histological examination in 79 patients, with an average of 1.5 biopsies/excisional biopsies per PWA. Indeed, 24 of the 79 patients had more than one biopsy/excisional biopsy (15 had two biopsies/excisional biopsies, 6 had three biopsies/excisional biopsies, and 3 had four biopsies/excisional biopsies). The mean age of the 79 patients was 24 ± 16.1 years (extremes: 7 and 75 years), and the sex-ratio (M/F) was 1. All of these 115 biopsies were cutaneous.

Histological examination led to a diagnosis in 110 cases (95.6%) and was inconclusive in 5 cases, including 4 cases of uncertain histological diagnosis and 1 case of nonspecific histological lesions.

Fourteen different histological diagnoses were made, with a frequency ranging from 0.9% (one case) to 26.9% (31 cases). The four most frequent diagnoses in descending order were basal cell carcinomas (BCCs) (31 cases; 26.9%), invasive squamous cell carcinomas (SCCs) or Bowen's disease (23 cases; 20%), keratosis (20 cases; 17.3%), and cysts (seven cases; 6.1%) (Table 1). The 54 cutaneous carcinomas were diagnosed in 33 (41.8%) of the 79 patients who underwent skin biopsies/excisional biopsies. The BCC/SCC ratio was 1.3. No cases of cutaneous melanoma had been diagnosed.

## 4. Discussion

In our study, 115 biopsies/excisional biopsies were performed in 79 PWA, an average of 1.5 biopsies/excisional biopsies per PWA. This rate is comparable to that found (1.6 biopsies per PWA) in Tanzania where 134 biopsies were performed on 86 PWA [2]. In Nigeria [9], 58 biopsies were taken from 30 PWA, corresponding to 1.9 PWA biopsies. These results show that suspicious skin lesions requiring skin biopsy are most often multiple in the same PWA, probably due to the effects of ultraviolet radiation. Moreover, the main reason for biopsies/excisional biopsies in PWA is especially when skin lesions are suspected to be malignant, as the practitioner's obsession is the early detection of these skin cancers in this population because of their very high frequency [9–12]. In the series of Enechukwu et al. [9], skin biopsy was performed on suspicion of premalignant or malignant lesions in 38% and 32% of cases, respectively.

Histological examination resulted in a histological diagnosis of certainty in 110 (95.6%) of the 115 biopsies/excisional biopsies in our study. In a French study on penile dermatosis in the general population [13], this histological examination led to a diagnosis in 85.5% of cases. Rajaratnam et al. [14] in Ireland found in the study of general population that, in 55% of cases of inflammatory dermatosis, histology led to a diagnosis of certainty, even without any clinical information provided to the pathologist. These results show the value of histology in dermatology for the diagnosis of skin diseases.

In our study, 33 (41.8%) of the 79 patients who had undergone biopsies/excisional biopsies had skin carcinomas (BCCs in 26.9% of cases and invasive or in situ SCC in 20% of cases). Other histological diagnoses were dominated by keratosis (17.3%), cysts (6.1%), hamartoma (6.2%), nodular folliculitis (6.1%), and benign squamous papillomas (6.1%). In the series of Enechukwu et al. [9], 18 (60%) of the 30 patients biopsied had skin carcinomas including BCC (37.9%), SCC (15%), basosquamous carcinoma (12%), and collision tumour (BCC and SCC, 3.4%). Other histological diagnoses were actinic keratosis (15%), nevi (12%), solar elastosis (1.2%), and psoriasis (1.7%). From these two studies, premalignant and malignant dermatosis were found to be the main histological diagnosis in PWA (46.9% in our series versus 83.3% in the Nigerian series [9]). These results confirm the impact of the sun, with a high prevalence of actinic damage and skin cancer. Indeed, the appearance of actinic lesions in PWA is not only related to age [4, 10] but also and especially to the hot and sunny climate [15, 16].

In our study, there was a predominance of BCC with a BCC/SCC ratio of 1.3. This predominance could be explained by the fact that, in our case, it is the dermatologist who goes to the PWA, which allows us to detect mild cases. In Nigeria [9], BCC was also the most frequent skin carcinoma (55%), followed by SCC (22%), basosquamous carcinoma (12%), and collision tumour (BCC and SCC, 3.4%). In Brazil [10], 62% were BCC, 51% were SCC, and 7% were melanoma. All three studies, including ours, were population-based studies. However, most of the African studies, all hospital series, show that SCC is the most common cancer in this population [2, 17–19]. Finally, melanoma is rare in PWA with a similar incidence in the general population [10], a tumour that was not by us and Enechukwu et al. [9].

Limitations of study: the main limitation of this study is that histological examinations were performed by general pathologists and not dermatopathologists. Nevertheless, in 95.6% of cases, the histological examination made a diagnosis.

## 5. Conclusion

The results of this study show that skin cancers represent the main histological diagnosis in PWA (46.9%) in Togo in 2019. The pattern of cutaneous malignancies in PWA shows the same trend as that seen in Caucasians with a predominance of basal cell carcinomas. The popularization and respect of photoprotection measures and systematic and regular examination of the skin of these PWA will allow early detection and management of these skin cancers.

## Figures and Tables

**Table 1:** Histological diagnosis in PWA in Togo in 2019.

Histological diagnostics	Number	(%)
Basal cell carcinomas	31	26.9
Squamous cell carcinomas (in situ and invasive)	23	20.0
Keratosis^*∗*^	20	17.3
Cysts (epidermal, synovial, and sebaceous)	7	6.1
Hamartomes (folliculo-sebaceous, verruca sebaceous, and angiocrine)	6	5.2
Nodular folliculitis	6	5.2
Benign squamous papillomas	6	5.2
Hyperplastic fleshy buds	4	3.5
Lipomes	2	1.7
Plantar wart	1	0.9
Psoriasis	1	0.9
Supposedly benign round cell tumour	1	0.9
Ulcerated benign lobular hemangioma	1	0.9
Boil	1	0.9
Nonspecific skin ulceration	1	0.9
Histological diagnosis uncertain	4	3.5
Total	115	100

^*∗*^Actinic (2); benign (14); HPV/wart (4).

## Data Availability

Extracted data are with the authors and available for sharing on request.

## Abbreviations

ANAT: National association of albinos of Togo
BCC: Basal cell carcinoma
NGO: Nongovernmental organization
PWA: Patients with albinism
SCC: Squamous cell carcinoma
SOTODERM: Togolese society of dermatology and sexually transmitted infections
UV: Ultraviolet.

## References

[1] Hong E. S., Zeeb H., Repacholi M. H. (2006). Albinism in Africa as a public health issue. *BMC Public Health*.

[2] Kiprono S. K., Chaula B. M., Beltraminelli H. (2014). Histological review of skin cancers in African albinos: a 10-year retrospective review. *BMC Cancer*.

[3] Sengupta M., Sarkar D., Mondal M., Samanta S., Sil A., Ray K. (2013). Analysis of MC1R variants in Indian oculocutaneous albinism patients: highlighting the risk of skin cancer among albinos. *Journal of Genetics*.

[4] Emadi S. E., Juma Suleh A., Babamahmoodi F. (2017). Common malignant cutaneous conditions among albinos in Kenya. *Medical Journal of the Islamic Republic Of Iran*.

[5] Okoro A. N. (1975). Albinism in Nigeria: a clinical and social study. *British Journal of Dermatology*.

[6] Luande J., Henschke C. I., Mohammed N. (1985). The Tanzanian human albino skin: natural history. *Cancer*.

[7] Van Der Westhuizen G., Beukes C. A., Green B., Sinclair W., Goedhals J., Goedhals J. (2015). A histopathological study of melanocytic and pigmented skin lesions in patients with albinism. *Journal of Cutaneous Pathology*.

[8] Perez M. I., Sanchez J. L. (1985). Histopathologic evaluation of melanocytic nevi in oculocutaneous albinism. *The American Journal of Dermatopathology*.

[9] Enechukwu N. A., Ogun G. O., Ezejiofor O. I. (2020). Histopathologic patterns of cutaneous malignancies in individuals with oculocutaneous albinism in anambra state, Nigeria: a paradigm swing?. *Ecancer Medical Science*.

[10] Marçon C. R., Moraes J. C., De Olivas Ferreira M. A. M., Oliari C. B. (2020). Dermatological and epidemiological profiles of patients with albinism in são paulo, Brazil, between 2010 and 2017: a cross-sectional study. *Dermatology*.

[11] Lookingbill D. P., Lookingbill G. L., Leppard B. (1995). Actinic damage and skin cancer in albinos in northern Tanzania: findings in 164 patients enrolled in an outreach skin care program. *Journal of the American Academy of Dermatology*.

[12] Kromberg J. G., Castle D., Zwane E. M., Jenkins T. (1989). Albinism and skin cancer in southern Africa. *Clinical Genetics*.

[13] Dauendorffer J.-N., Renaud-Vilmer C., Bagot M., Cavelier-Balloy B. (2012). Intérêt de la biopsie cutanée dans le diagnostic des dermatoses péniennes. *Annales de Dermatologie et de Vénéréologie*.

[14] Rajaratnam R., Smith A. G., Biswas A., Stephens M. (2009). The value of skin biopsy in inflammatory dermatoses. *The American Journal of Dermatopathology*.

[15] Wright C. Y., Norval M., Hertle R. W. (2015). Oculocutaneous albinism in sub-saharan Africa: adverse sun-associated health effects and photoprotection. *Photochemistry and Photobiology*.

[16] Wright C. Y., Du Preez D. J., Millar D. A., Norval M. (2020). The epidemiology of skin cancer and public health strategies for its prevention in southern africa. *International Journal of Environmental Research and Public Health*.

[17] Mabula J. B., Chalya P. L., Mchembe M. D. (2012). Skin cancers among albinos at a university teaching hospital in northwestern Tanzania: a retrospective review of 64 cases. *BMC Dermatology*.

[18] Awe O., Azeke T. (2018). Cutaneous cancers in Nigerian albinos: a review of 22 cases. *Nigerian Journal of Surgery*.

[19] Opara K. O., Jiburum B. C. (2010). Skin cancers in albinos in a teaching hospital in eastern Nigeria—presentation and challenges of care. *World Journal of Surgical Oncology*.

